# Sporadic adult-onset neuronal intranuclear inclusion disease without high-intensity signal on DWI and T2WI: a case report

**DOI:** 10.1186/s12883-022-02673-7

**Published:** 2022-04-22

**Authors:** Hongfen Wang, Feng Feng, Jiajin Liu, Jianwen Deng, Jiongming Bai, Wei Zhang, Luning Wang, Baixuan Xu, Xusheng Huang

**Affiliations:** 1 Department of Neurology, First Medical Center, Chinese PLA General Hospital, Beijing, China; 2 Department of Neurology, PLA Rocket Force Characteristic Medical Center, Beijing, China; 3 Department of Nuclear Medicine, First Medical Center, Chinese PLA General Hospital, Beijing, China; 4 Department of Neurology, Peking University First Hospital, Beijing, China; 5 Beijing Key Laboratory of Neurovascular Disease Discovery, Beijing, China; 6 Department of Neurology, Second Medical Center, Chinese PLA General Hospital, Beijing, China

**Keywords:** Neuronal intranuclear inclusion disease, Diffusion-weighted imaging, *NOTCH2NLC* gene, Skin biopsy, Case report

## Abstract

**Background:**

Neuronal intranuclear inclusion disease (NIID) is a rare neurodegenerative disease characterized by eosinophilic hyaline intranuclear inclusions in cells in the central and peripheral nervous system. High-intensity signal in the corticomedullary junction on diffusion-weighted imaging (DWI) is supportive to the diagnosis of NIID. We describe a patient with sporadic adult-onset NIID but without any high-intensity signal on DWI and T2-weighted imaging (T2WI).

**Case presentation:**

A 58-year-old woman without special family history developed mild persistent tremor in the right hand and deteriorated 2 years later. At 60 years of age, the patient began to conceive the bank, police and internet being deceptive, further presented apathy and confusion after two and a half years, as well as fabrication of non-existent things. Despite the treatment of antipsychotic drugs due to a diagnosis of mental disorder, the patient appeared weakness in the right limbs. Neurological examination revealed mutism, resting tremor, cogwheel-like rigidity in upper limbs, and weakness in all limbs. Brain magnetic resonance imaging displayed no cerebral atrophy initially but atrophy of frontal, temporal and parietal lobes 5 years later. No any high-intensity signal on DWI and T2WI was revealed. However, hypometabolism in the cortexes with atrophy and the right putamen nucleus were showed on ^18^F-fluoro-deoxy-glucose positron emission tomography/magnetic resonance. On the basis of 107 GGC repeats (normal number <40) in *NOTCH2NLC* gene and intranuclear inclusions with p62 immunoreactivity in the adipocyte of cutaneous sweat duct by skin biopsy, NIID was finally diagnosed. The symptomatic treatment was given but the patient had no evident improvement.

**Conclusions:**

Our case highlights that despite the lack of high-intensity signal on DWI and T2WI, NIID is still considered for patients with parkinsonism and mental impairment.

## Background

Neuronal intranuclear inclusion disease (NIID) is a neurodegenerative disease characterized by eosinophilic hyaline intranuclear inclusions in cells in the central and peripheral nervous system as well as visceral organs. NIID is categorized into infantile, juvenile, and adult variants according to age of onset, and is also divided into familial and sporadic cases [1]. Due to highly heterogeneous clinical manifestations, ante-mortem diagnosis of NIID is inevitably a challenge. Since 2011, the positive hyaline intranuclear inclusions in skin biopsy has been used for the diagnosis of NIID [2], and the confirmed cases have been increasing. In 2019, abnormal trinucleotide (GGC) repeat expansion in *NOTCH2NLC* gene was found to be related with NIID [3–6].

High-intensity signal in the corticomedullary junction on diffusion-weighted imaging (DWI) is a strong indicator for the diagnosis of adult-onset NIID, and reported in all of the sporadic adult-onset NIID cases and most of the familial NIID cases [7]. However, DWI hyperintensities in the corticomedullary junction were not detected in a few patients with sporadic adult-onset NIID [8–10], while the mild, remarkably progressive or focally reversible leukoencephalopathy were found on T2-weighted imaging (T2WI). Herein, we report a patient with adult-onset sporadic NIID who had no any high-intensity signal on DWI and T2WI.

## Case presentation

A 58-year-old Chinese Han woman was admitted to our hospital, with a complaint of subsequent tremor, mental disorder and limbs weakness since she was 53 years old. The patient initially presented mild persistent tremor in the right hand but accepted no special treatment. Two years later, the tremor deteriorated to affect eating. At the same time, the patient began to conceive the bank, police and internet being deceptive, but still accepted no consultation. After two and a half years, apathy, diminished social interest, confusion, fabrication of non-existent things, oligologia, and decreased appetite, appeared in succession. One month later, the patient was diagnosed with mental disorder in a psychiatric clinic and given olanzapine 10 mg and oxazepam 7.5 mg one time every night and aripiprazole 2.5 mg one time every day to improve the psychiatric symptoms, as well as given trihexyphenidyl 2.5 mg three times every day to alleviate tremors. Considering the diagnosis of Parkinson’s disease (PD), madopar was also prescribed with a dosage of 125 mg three times every day but stopped 4 days later due to the emergence of unsteadiness in standing. About one and a half months later, weakness in the right limbs appeared and gradually deteriorated to stand with assistance. After 2 months, olanzapine, oxazepam, aripiprazole, and trihexyphenidyl were all stopped. During treatment of the above drugs, slow response, occasional urinary and fecal incontinence, sleep reversal, and mutism emerged. One month later, the patient came to our clinic for further consultation. No fever, seizure, infection and dysphagia were reported by the caregivers, except for dysphonia.

Neurological examination revealed mutism, resting tremor and cogwheel-like rigidity in upper limbs, weakness in all limbs especially in the right lower limb, decreased deep tendon reflexes except the right upper limb, positive jaw jerk and palmomaxillary reflex, but no orthostatic hypotension and pathological reflexes. The Mini Mental State Examination (MMSE) and other neurological examination could not be accomplished by her conditions.

Hematological, biochemical and immune-related blood tests were normal results. Normal cell count, glucose level and immune-related indexes except mildly increased protein level (530.0 (150–400) mg/L) were detected in cerebrospinal fluid by lumbar puncture. Parameters related with tumor and paraneoplastic syndrome in blood and cerebrospinal fluid were all negative. Twenty-four hours ambulatory electroencephalograph revealed no abnormality. Nerve conduction study showed decreased compound muscle action potential amplitude in bilateral peroneal nerves. Needle electromyography examination detected chronic denervation potential in right tibialis anterior.

**Fig. 1:**
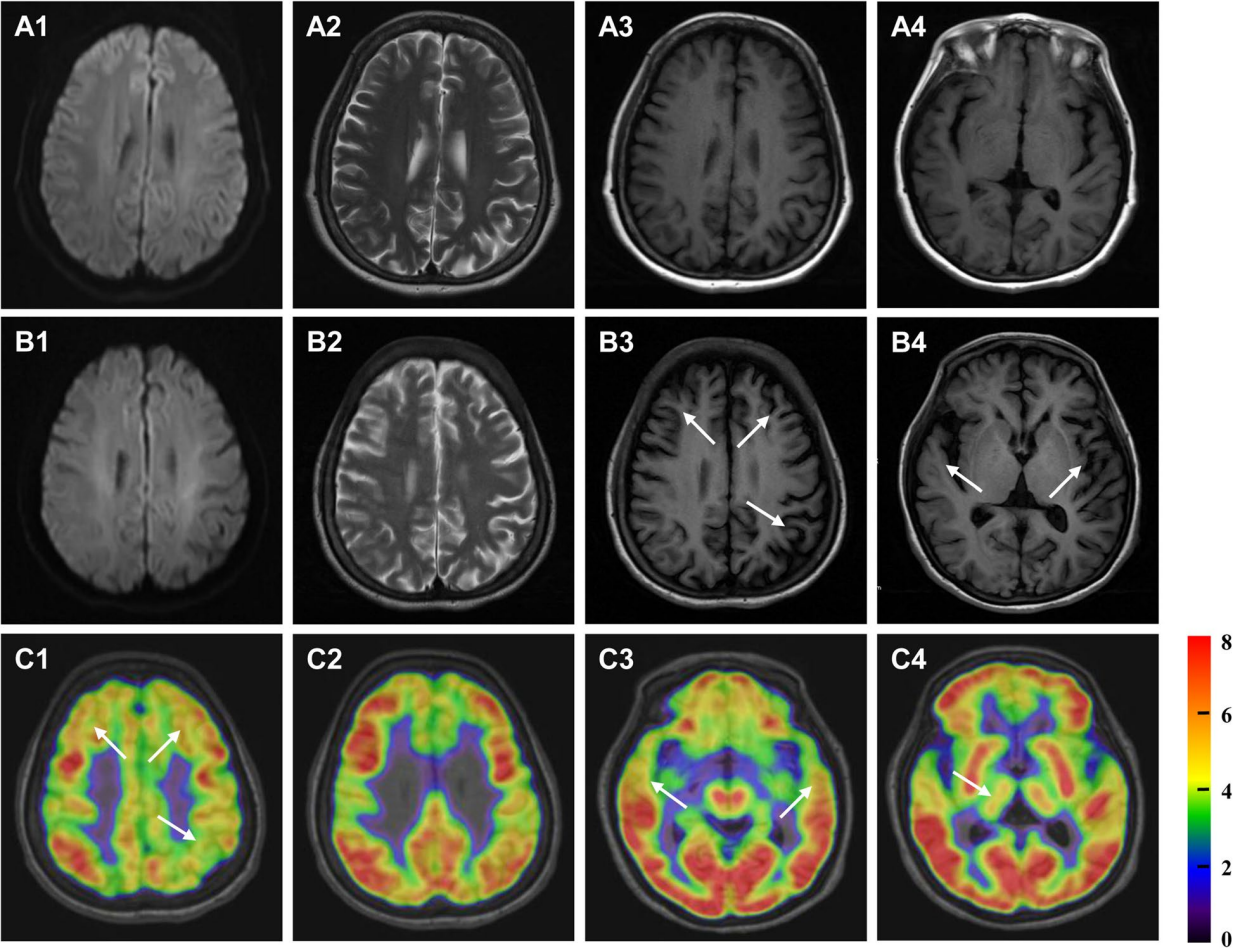
Brain MRI and ^18^F-FDG PET/MR examination. **A1**-**A4** First brain MRI at onset showed no apparent cerebral atrophy and abnormal signals on DWI and T2WI. **B1**-**B4** Second brain MRI five years later after onset, revealed atrophy of left parietal lobe, bilateral frontal lobes, and bilateral temporal lobes, but still without high-intensity lesions on DWI and T2WI. **C1**-**C4** ^18^F-FDG PET/MR showed hypometabolism of left parietal cortex, bilateral medial prefrontal cortexes (**C1**, **C2**), bilateral anterior temporal cortexes (**C3**), and right putamen nucleus (**C4**)

**Fig. 2:**
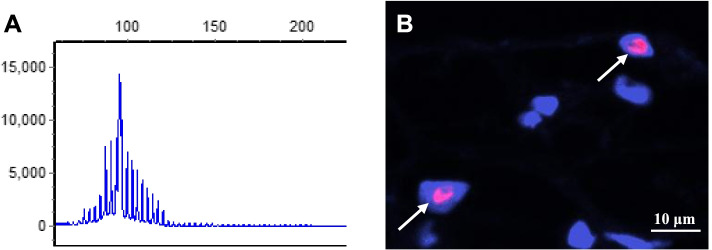
Results of gene detection and skin biopsy. **A** Repeat-primed PCR showed 107 GGC repeats in *NOTCH2NLC* gene. **B** p62 fluorescence staining of skin biopsy with punch. The red signal indicated intranuclear inclusions in lipocyte of sudoriferous duct

The initial brain magnetic resonance imaging (MRI) scanned at the age of 53 years old, and displayed no cerebral atrophy and hyperintensities on DWI and T2WI (Fig. 1 A1-A4). However, in 58 years old, brain MRI revealed atrophy of frontal, temporal and parietal lobes, but still without high-intensity signals on DWI and T2WI (Fig. 1 B1-B4). At the same time, ^18^F-fluoro-deoxy-glucose (FDG) positron emission tomography/magnetic resonance (PET/MR) showed hypometabolism in the corresponding cortexes with atrophy, and in the right putamen nucleus (Fig. 1 C1-C4). The result of whole exome sequencing was negative. Detection of GGC repeats number in *NOTCH2NLC* gene showed 107 (normal number <40), an abnormal expansion (Fig. 2A). Skin biopsy with punch from 10 cm above ankle was carried out, and displayed intranuclear inclusions with p62 immunoreactivity in the adipocyte of cutaneous sweat duct (Fig. 2B). Finally, the diagnosis of NIID was made. Due to no effective drugs for NIID at present, only the symptomatic treatment was given. The patient had no evident improvement half a year later.

## Discussion

In the current study, we report a sporadic adult-onset NIID case, presenting with parkinsonism and mental impairment. Skin biopsy and gene detection demonstrated the diagnosis of NIID.

High-intensity signal in the corticomedullary junction on DWI, being called ‘ribbon sign’ or ‘diaper sign’, was presented in 100% patients with sporadic NIID while in 81.8% patients with hereditary NIID [7], and was found a spread pattern along the corticomedullary junction [7, 11, 12]. However, Cupidi et al. described mild leukoaraiosis but without high-intensity signal in the corticomedullary junction on DWI in a sporadic adult-onset NIID patient, presenting with dementia, behavioral disturbances and parkinsonism [8]. The other sporadic adult-onset NIID patient showed remarkable progressive leukoencephalopathy and encephalatrophy, but no DWI hyperintensities in the corticomedullary junction [9]. Another sporadic adult-onset NIID patient was reported by Cao et al., who only showed focal reversible leukoencephalopathy but no DWI high signals [10]. Being diagnosed with sporadic adult-onset NIID, our case was revealed no any high-intensity lesions on DWI and abnormal signals on T1-weighted imaging (T1WI) or T2WI by two brain MRI examinations. The current case implied that high signal in the corticomedullary junction on DWI and leukoencephalopathy on T2WI can be concurrently absent in sporadic adult-onset NIID. Furthermore, brain atrophy was also found in all the reported sporadic adult-onset NIID patients despite the lack of DWI hyperintensities in the corticomedullary junction [8–10]. The mild and diffuse brain atrophy was reported by Dong et al. [9], while cerebral atrophy was described without details by Cao et al. [10]. The prominent atrophy in the frontal and temporal lobes, was found by Cupidi et al. [8] and in our case.

In particular, DWI showed a small high-intensity lesion in the right frontal corticomedullary junction until 7 years after the first episode of recurrent vomiting in a patient with NIID [13]. Further, a 62-year-old woman with NIID was reported a disappearance of DWI hyperintensities in the corticomedullary junction after 5 years despite disease progression [14]. Additionally, another case of NIID showed no abnormal brain lesions at the initial stage, but displayed the progressive linear hyperintensities in the frontal corticomedullary junction, and the reversible high-intensity signal in the occipital lobe on DWI in the duration [15]. Similarly, the high-intensity signals on DWI were absent at the onset, but presented in the corticomedullary junction area 6 years later, and ultimately disappeared 8 years after onset in a NIID patient [16]. Thus, for our patient, we speculate the brain DWI high-intensity signals might have disappeared in the disease course or still unreach the time of emergence. The follow-up containing brain MRI examination, is definitely needed in the future. Moreover, subcortical DWI high-intensity signals in NIID were reported strongly correlated with pathological spongiotic changes [17]. However, the absence of pathological diffuse myelin pallor and spongiotic changes were demonstrated in the NIID case without typical DWI abnormalities [8], which implying that the dynamic imaging features of NIID may be due to pathologic alterations in different stages.

Despite the majority of sporadic NIID cases showed regionally decreased cerebral blood flow of the frontal, parietal, precuneous, and posterior cingulate cortices on single-photon computed emission tomography (SPECT) [7, 18, 19], a few cases carried out positron emission tomography/computed tomography (PET/CT) with ^18^F-FDG to explore the brain metabolism. In a sporadic adult-onset NIID case predominantly with dementia and gait disturbance, ^18^F-FDG PET/CT demonstrated glucose hypometabolism in bilateral cerebral hemisphere, especially in the frontoparietal cortex, wider than the range of leukoencephalopathy on MRI [20]. The ^18^F-FDG PET/CT of a familial adult-onset NIID patient with dementia and Parkinson’s syndrome showed decreased metabolism in the left frontal lobe and right parietal lobe [21]. Furthermore, PET/MR demonstrated decreased metabolic activity in the bilateral frontal lobes and part of the temporal lobe in a familial adult-onset patient with dementia and paroxysmal encephalopathy [10]. In our case, PET/MR with ^18^F-FDG also demonstrated glucose hypometabolism in the left parietal, bilateral medial prefrontal, and bilateral anterior temporal cortexes, alongside corresponding atrophy. Although SPECT showed normal uptake in the thalamus and basal ganglia in a NIID patient presenting with resting tremor but no other signs of parkinsonism [22], our case revealed hypometabolism of right putamen nucleus, which partly explaining her resting tremor and increased muscle tone.

## Conclusions

For the first time in the literatures, we describe a patient with sporadic adult-onset NIID who displayed no high-intensity signal on DWI and T2WI. On account of the apparent mental impairment is not always captured in the duration of PD or Parkinson’s syndrome, neurological clinicians are supposed to consider the diagnosis of NIID for patients with mental impairment and parkinsonism even lack of the high-intensity signal on DWI and T2WI.

## Data Availability

The data used within this article will be made available from the corresponding authors on reasonable request.

## Abbreviations

DWI: Diffusion-weighted imaging
FDG: Fluoro-deoxy-glucose
MMSE: Mini Mental State Examination
MRI: Magnetic resonance imaging
NIID: Neuronal intranuclear inclusion disease
PD: Parkinson’s disease
PET/CT: Positron emission tomography/computed tomography
PET/MR: Positron emission tomography/magnetic resonance
SPECT: Single-photon computed emission tomography
T1WI: T1-weighted imaging
T2WI: T2-weighted imaging
